# The intersection of near-death experiences (NDEs) and traumatic brain injury (TBI): neurobiological, phenomenological, and creative implications

**DOI:** 10.3389/fnhum.2025.1649513

**Published:** 2025-11-28

**Authors:** Diego Iacono, Gloria C. Feltis

**Affiliations:** 1 Neuropathology Research, Biomedical Research Institute of New Jersey (BRInj), Cedar Knolls, NJ, United States; 2 Neuroscience Research, MidAtlantic Neonatology Associates (MANA), Department of Pediatrics, Atlantic Health System (AHS), Morristown, NJ, United States; 3 Neurodevelopmental Research Lab, Biomedical Research Institute of New Jersey (BRInj), Cedar Knolls, NJ, United States; 4 Department of Neurology, Thomas Jefferson University, Philadelphia, PA, United States; 5 Library and Information Science, Bethesda, MD, United States

**Keywords:** near-death experience (NDE), traumatic brain injury (TBI), psychedelic experience, neurobiology, neuropharmacology, human creativity, personality and spiritual changes

## Abstract

Traumatic brain injury (TBI) and near-death experiences (NDEs) represent profound disruptions in brain function, often associated with dramatic changes in consciousness, self-perception, and behavior. While these events are typically studied for their pathological consequences, a growing body of evidence suggests they may also trigger unexpected cognitive and creative enhancements in a subset of individuals. We explore the intersection between TBI, NDEs, and the emergence of heightened artistic expression, integrating findings from neuroanatomy, neuropathology, genetics, and phenomenology. We examine how alterations in key brain networks - such as the default mode network, frontoparietal circuits, and limbic regions - may underpin shifts in self-awareness, emotion processing, and symbolic thinking, which are frequently reported after NDEs or severe brain trauma. Additionally, we discuss the potential role of neuroplasticity, diaschisis, and compensatory reorganization in facilitating novel patterns of cognition and creative output following injury. Genetic factors potentially influencing susceptibility to such transformations are considered, alongside evolutionary perspectives on why these rare post-injury enhancements may occur. By synthesizing clinical cases, neuroscientific studies, and first-person accounts, we argue that certain brain injuries and altered states of consciousness can function as catalysts for reconfiguration of cognitive and emotional systems, leading to emergent artistic abilities or intensified creative insight. These phenomena challenge traditional dichotomies between damage and function, pathology and creativity, and invite new frameworks for understanding the plastic potential of the human brain. This overview-hypothesis driven article aims to contribute to a deeper understanding of how the mind and brain interact under extreme conditions and how these interactions may unlock hidden reservoirs of human potential. The paper highlights the need for systematic research into post-traumatic and NDE-related cognitive changes, not only to improve clinical outcomes but also to broaden our knowledge of human consciousness and creativity.

## Introduction

Traumatic brain injury (TBI) remains one of the leading causes of death and disability globally, affecting millions of people each year. The consequences of TBI can be far-reaching, leading to lasting cognitive, emotional, and neurological deficits. However, in some cases, TBI can also give rise to extraordinary phenomena, including near-death experiences (NDEs). Indeed, while systematic data is limited, the link has been empirically documented. Hou et al. (2013), in the only study to date specifically investigating NDEs in a severe TBI cohort, reported their prevalence and characteristics, providing a crucial foundation for this line of inquiry. For the purposes of this review, we needed to provide an explicit definition of Traumatic Brain Injury (TBI), which we defined as physical, structural trauma to the head and brain (e.g., from falls, vehicular accidents, or blunt force). We differentiated this from NDEs precipitated by other causes (e.g., primary cardiac arrest), which may converge on a final common pathway of hypoxia but lack the unique upstream mechanical insult and diffuse axonal injury characteristic of TBI. On the other side, the origin of NDEs remains a topic of intense debate. While some researchers highlight the transcendental aspects that challenge conventional models, there is a growing body of scientific research suggesting that NDEs are rooted in the complex neurobiology of the brain during extreme states of dysfunction, such as those triggered by TBI (e.g., Xu et al., 2023; Borjigin et al., 2013). NDEs are subjective experiences, often occurring in individuals who have been close to death or have experienced extreme physiological distress, such as during a life-threatening injury or medical procedure. These experiences can include phenomena such as out-of-body experiences, visions of light, encounters with deceased loved ones, and profound spiritual revelations. Although NDEs have traditionally been considered a spiritual or metaphysical phenomenon, there is a growing body of scientific research suggesting that NDEs are rooted in the complex neurobiology of the brain, particularly during extreme states of dysfunction such as those triggered by TBI (Greyson, 2000; Parnia et al., 2014).

The intersection of TBI and NDEs presents a unique opportunity to examine the intricate neurobiological and psychological consequences of severe brain injury, with profound implications for our understanding of consciousness, self-awareness, and the brain’s remarkable capacity for both recovery and transformation. This paper seeks to delve deeper into the neurobiological mechanisms underlying NDEs in TBI survivors, examining clinical case studies, advanced neuroimaging findings, genetic and epigenetic insights, the role of various neurobiological perturbations, and the implications of these extraordinary experiences for creativity and artistic expression post-injury. Furthermore, we will explore the theoretical relevance of emerging neuromodulatory and pharmacological interventions, such as Deep Brain Stimulation (DBS), Transcranial Magnetic Stimulation (TMS), and ketamine, in understanding and potentially influencing altered states of consciousness akin to NDEs.

## TBI as a potential trigger for NDEs

Near-death experiences are frequently reported in individuals who have sustained severe TBIs, particularly those who are at significant risk of death or undergo prolonged periods of unconsciousness, such as coma. The probability and phenomenology of experiencing an NDE appear to correlate with the severity of the brain injury and the duration of consciousness impairment. Clinical reports of NDEs following TBI date back several decades, with some of the earliest documented cases emerging from survivors of car accidents, falls, and other traumatic incidents that induce acute and severe physiological stress on the brain (Moody, 1975). These early observations provided anecdotal evidence that extreme physiological states could precipitate profound subjective experiences.

For example, *Case 1*: Mary, a patient who survived a catastrophic motor vehicle accident, describes a vivid NDE that occurred while she was in a deep coma. She reported an explicit sensation floating above her physical body and observing the accident scene from an external, disembodied vantage point, followed by a profound feeling of being surrounded by an overwhelming, benevolent light. Mary’s experience mirrors many of the classic, universally reported features of NDEs, including the quintessential out-of-body experience (OBE), which is widely hypothesized to be linked to disrupted processing of sensory input and self-awareness within the brain’s proprioceptive and vestibular systems. This case strongly supports the hypothesis that severe TBI and associated physiological insults like hypoxia (oxygen deprivation) may trigger NDE-like experiences by directly altering brain function in critical areas related to self-perception (Martial et al., 2024). Another compelling example is *Case 2*: Tony Cicoria, a renowned orthopedic surgeon, who suffered a lightning strike that precipitated a profound NDE. During his recovery phase, Tony unexpectedly developed an intense, consuming passion for classical music and began composing complex piano pieces, despite having no prior musical training or significant interest. This case underscores the inherent complexity and multifaceted nature of NDEs, which can be accompanied by unpredictable and profound neurological effects, including the spontaneous emergence of artistic talents that were previously dormant or entirely absent prior to the injury (Sacks, 2007). Such cases challenge conventional notions of brain injury outcomes, highlighting a potential for unexpected cognitive and creative transformations.

## Neurobiological theories: mechanisms underlying NDEs in TBI

A rapidly growing body of contemporary research robustly suggests that NDEs are not merely ephemeral spiritual phenomena but may instead result from complex neurobiological processes specifically triggered by extreme physical or psychological stress. This stress can manifest in various forms, including profound hypoxia, cerebral ischemia (reduced blood flow), electrolyte imbalances, and severe brain dysfunction directly resulting from TBI. Several interconnected brain regions have been consistently implicated in the onset and phenomenology of NDEs, particularly those intricate areas involved in the highly integrated functions of self-awareness, body perception (somatosensory processing), and emotional regulation.

### Hypoxia and glutamate excitotoxicity

The pervasive phenomenon of hypoxia - a critical reduction in the supply of oxygen to brain tissues - is an extremely common and detrimental consequence in individuals with severe TBI, particularly those who experience prolonged periods of loss of consciousness or enter a comatose state. Hypoxia is known to differentially affect specific, metabolically vulnerable regions of the brain, such as the temporal lobes, the precuneus, and the temporo-parietal junction (TPJ) (Eddy, 2016; Graham, 2004). These regions are extensively involved in multifaceted aspects of the sense of self, spatial orientation and navigation, and the dynamic maintenance of consciousness itself (Blanke et al., 2002; Blanke and Metzinger, 2009). Studies have consistently demonstrated that acute or chronic disruptions in these highly sensitive cortical areas, which are particularly susceptible to even minor reductions in oxygen levels due to their high metabolic demand, can directly lead to profound alterations in body perception, including feelings of disembodiment or a complete dissociation from the physical body–features commonly and vividly described in NDEs (Bünning and Blanke, 2005). For instance, sophisticated functional neuroimaging studies, employing techniques such as fMRI and PET scans, have compellingly demonstrated that targeted electrical or magnetic stimulation of the TPJ in healthy individuals can reliably induce sensations strongly reminiscent of an out-of-body experience (OBE) (van Elk et al., 2017). This robust experimental evidence strongly suggests that direct structural damage or acute functional disruption to this critical region during TBI could potentially result in the spontaneous and vivid emergence of such dissociative experiences in the immediate or prolonged aftermath of a traumatic event. The subsequent altered neuronal excitability, synaptic plasticity, and network connectivity induced by excitotoxicity could directly contribute to the unusual perceptual and cognitive states commonly reported in NDEs, such as accelerated thought processes, vivid memories, and profound insights. More mechanistically, we hypothesize that this glutamatergic over-activity creates a “network storm” in memory (hippocampus) and cognitive (PFC) circuits by lowering the threshold for neuronal firing. This cascade of uncontrolled activation is experienced phenomenologically as accelerated thoughts and vivid, spontaneous memory recall.

## Neurotransmitter dysregulation and NDEs

Beyond hypoxia, widespread neurotransmitter dysregulation plays an unequivocally critical and multifaceted role in the onset of altered states of consciousness, including the complex phenomena reported during NDEs. Specifically, the acute and chronic dysregulation of crucial signaling pathways involving dopamine, serotonin, and glutamate can profoundly contribute to altered perceptions, vivid hallucinations, and profound dissociative experiences (Strassman, 2001). TBI, through various primary and secondary injury mechanisms, can induce significant and often lasting disruptions in the delicate balance of these vital neurotransmitter systems, thereby directly leading to the kind of dramatic perceptual and cognitive shifts that fundamentally characterize NDEs. The specific mechanisms of neurotransmitter disruption are crucial for a comprehensive understanding. For example, excitotoxicity, primarily mediated by an excessive and uncontrolled release of the excitatory neurotransmitter glutamate, coupled with the pathological overactivation of its primary receptor, the N-methyl-D-aspartate (NMDA) receptor, represents a major and devastating secondary injury mechanism following TBI (Hossmann, 1994). This cascade of events leads to a catastrophic and overwhelming influx of calcium ions into neurons, triggering severe mitochondrial dysfunction, massive oxidative stress, and widespread neuronal death. The subsequent altered neuronal excitability, synaptic plasticity, and network connectivity induced by excitotoxicity could directly contribute to the unusual perceptual and cognitive states commonly reported in NDEs, such as accelerated thought processes, vivid memories, and profound insights. Furthermore, the endogenous opioid system is a compelling candidate for mediating some aspects of NDE phenomenology. Severe physical or psychological stress, including that experienced during life-threatening trauma, is known to trigger the rapid and massive release of endogenous opioid peptides (e.g., endorphins, enkephalins) (Akil et al., 1984). This surge of opioids can induce profound feelings of peace, euphoria, analgesia, and emotional detachment, all of which are frequently reported during NDEs (Jansen, 1997). The activation of mu-opioid receptors could contribute to the sense of wellbeing and transcended pain.

The serotonergic system, especially the 5-HT2A receptors, is also intricately involved in modulating states of consciousness and is the primary target for many classical hallucinogens (Nichols et al., 2017). TBI-induced alterations in serotonin synthesis, release, reuptake, or receptor sensitivity could significantly contribute to the vivid visual and auditory hallucinations, encounters with deceased loved ones, and spiritual revelations characteristic of NDEs. Mechanistically, we propose that TBI-induced diffuse axonal injury can directly damage the serotonergic axons originating in the brainstem (raphe nuclei). This physical shearing, followed by secondary inflammation, can disrupt serotonin synthesis and release, leading to acute dysregulation of 5-HT2A receptors critical for perception and emotion. The widespread distribution of serotonergic neurons, originating from the brainstem raphe nuclei, and their projections throughout the cortex and limbic system, mean that even diffuse axonal injury (DAI) common in TBI can profoundly impact this system.

## Pharmacological models: ketamine and DMT

Ketamine is a dissociative anesthetic and increasingly recognized as a rapid-acting antidepressant. Its primary mechanism of action involves non-competitive antagonism of the NMDA receptor, a type of glutamate receptor (Zarate et al., 2006). At sub-anesthetic doses, ketamine produces profound dissociative states, altered perceptions, vivid hallucinations, and a unique sense of ego dissolution or “mystical experience.”

Ketamine’s ability to induce dissociative states strongly aligns with the hypothesis that alterations in glutamatergic signaling, particularly involving NMDA receptors, are central to the dissociative aspects of NDEs (Jansen, 1997; Krystal et al., 1994). TBI often leads to excitotoxicity mediated by excessive glutamate and NMDA receptor activation, but the complex interplay of overactivation, subsequent downregulation, or receptor damage could produce various states, including dissociation. Ketamine provides a pharmacological model to study these effects in a controlled setting. The out-of-body sensations, feelings of unreality, and altered body schema reported with ketamine bear striking resemblances to NDE reports. Higher doses of ketamine can induce a profound sense of ego dissolution, feelings of unity, and a sense of transcending physical boundaries, which are core features of many NDEs and mystical experiences (Fritz et al., 2025). This suggests that pharmacological modulation of NMDA receptors can directly access neural pathways that mediate these transcendental states, offering a unique opportunity to study their neurochemical basis. These phenomenological parallels have been systematically explored (e.g., Corazza, 2008) and offer a unique opportunity to study their neurochemical basis.

A significant and parallel hypothesis, which has been a notable omission, is the endogenous DMT model. The profound phenomenological overlap between NDEs and experiences induced by N,N-dimethyltryptamine (DMT) has been extensively documented (Luke, 2024). Building on this, Frecska et al. (2013) proposed that severe physiological stress or trauma, such as that incurred during a TBI, could trigger a protective, endogenous release of DMT. This release could drive the acute phenomenology of the NDE and contribute to subsequent neuroplastic and healing processes, particularly given DMT’s known neuroprotective effects (Szabo et al., 2016).

## Neuropathological and neuroanatomical insights into TBI-induced NDEs

Traumatic brain injury fundamentally disrupts brain integrity through both immediate primary mechanical damage (e.g., contusions, lacerations, diffuse axonal injury) and a complex array of insidious secondary processes that unfold over hours, days, and even weeks post-injury. These secondary mechanisms include but are not limited to neuroinflammation, ischemia, excitotoxicity, and proteinopathy. Critically, these pathological processes preferentially converge on and severely compromise brain regions intimately implicated in the generation and maintenance of consciousness and self-processing. These include the temporo-parietal junction (TPJ), the precuneus, the medial prefrontal cortex (mPFC), and the posterior cingulate cortex (PCC) all key nodes within the brain’s highly integrated network of self-awareness and conscious experience.

## Cortical disintegration and network collapse

Advanced neuroimaging techniques, such as functional MRI (fMRI) and diffusion tensor imaging (DTI), have provided compelling evidence that functional disintegration and altered structural connectivity within the default mode network (DMN)–a crucial resting-state network vital for self-referential thought, introspection, and episodic memory–are significantly correlated with the unique of consciousness and states possibly with the phenomenology of NDEs (Tagliazucchi and van Someren, 2017). While direct imaging during an NDE is not available, we hypothesize - drawing parallels from related states like psychedelic experiences (Carhart-Harris et al., 2016) - that DMN disintegration may correlate with NDE phenomenology. Acute TBI-induced network collapse, or alternatively, aberrant alterations in connectivity both within and between major intrinsic brain networks, including the DMN, the salience network (SN), and the central executive network (CEN), may facilitate a unique and transient state of neural decoupling. This decoupling could mimic the profound dissociative experiences and the widely reported floating or levitation sensations often experienced during NDEs. Specifically, we hypothesize this refers to the decoupling of the temporo-parietal junction (TPJ) from the rest of the DMN. Given that the TPJ is critical for multisensory integration and self-location processing, its disruption (e.g., via hypoxia) is believed to result in a failure to integrate bodily and spatial information, leading directly to the specific phenomenological experience of an out-of-body sensation (Blanke and Arzy, 2005). The SN, which is pivotal for detecting and integrating salient internal (e.g., pain, hunger) and external stimuli (e.g., sudden sounds, visual cues), and the CEN, which is critically involved in high-level cognitive control, working memory, and goal-directed behavior, are also profoundly affected in TBI (Bonnelle et al., 2012). Disruptions in these networks can lead to a fundamental breakdown of typical reality construction and perception, promoting a shift toward more introspective, hallucinatory, or otherwise anomalous states of consciousness. The degree of disruption and the specific network nodes affected may dictate the individual characteristics of an NDE.

## Secondary injury mechanisms and long-term changes

We believe that it is critical to distinguish the timing of these mechanisms. We now explicitly state that primary insults (e.g., acute hypoxia, excitotoxicity) are hypothesized to trigger the acute NDE experience itself. In contrast, secondary changes, which occur over hours to weeks, are reframed here as mechanisms for the long-term psychological and creative after-effects.

## Hypoxia-sensitive structures and mitochondrial dysfunction

The hippocampus, retrosplenial cortex, and thalamus–neural structures characterized by exceptionally high metabolic demands and consequently high oxygen consumption–are particularly vulnerable to hypoxia and ischemia following TBI. These regions are indispensably critical for temporal awareness, the encoding and retrieval of emotional memory, and spatial orientation and navigation. Consistently, studies have demonstrated that altered perfusion (blood flow) and structural integrity in these areas are strongly associated with the occurrence of out-of-body experiences (OBEs) and vivid life review phenomena during NDEs (Blanke and Arzy, 2005).

Crucially, mitochondrial dysfunction is a pervasive and devastating consequence of TBI, representing a cornerstone of secondary brain injury. Traumatic insults trigger a complex cascade of events, including pathological calcium overload within neurons, massive generation of reactive oxygen species leading to oxidative stress, and widespread lipid peroxidation of cell membranes (Hiebert et al., 2015). All these factors synergistically compromise mitochondrial integrity, morphology, and function, leading to a severe and widespread energy crisis within neural cells. This energy deficit critically impairs ATP-dependent processes such as ion pump function (leading to sustained depolarization), and neurotransmitter reuptake mechanisms, thereby contributing significantly to the widespread neuronal dysfunction and cell death observed. The profound energy deficit disproportionately impacts highly active and metabolically demanding brain regions, exacerbating the effects of hypoxia and contributing profoundly to the altered states of consciousness characteristic of NDEs. Furthermore, impaired mitochondrial function can lead to an amplified production of reactive oxygen species, which oxidative stress can, in turn, damage neuronal structures, particularly myelin, and disrupt key neuroplasticity pathways like BDNF-TrkB, thereby shaping the long-term recovery environment and potentially contributing to lasting psychological shifts, and ultimately, causing further irreparable damage to neuronal structures and potentially altering crucial signaling pathways, including those involved in consciousness and perception.

## Neuropathology of altered consciousness: neuroinflammation, blood-brain barrier (BBB) disruption, and glial activation

In addition to acute functional changes and energy deficits, TBI frequently induces persistent proteinopathies, such as hyperphosphorylated tauopathy and TDP-43 aggregation, which are typically associated with neurodegenerative diseases (Mckee and Daneshvar, 2015). These chronic pathologies may progressively alter cortical excitability, synaptic plasticity, and memory processing, potentially exacerbating dysregulated states of awareness and fragmented but vivid recollections during NDEs or in their aftermath. A critical and sustained secondary injury mechanism post-TBI is neuroinflammation, a complex immunological response characterized by the rapid activation of resident brain immune cells, specifically microglia and astrocytes, and the subsequent pathological infiltration of peripheral immune cells (e.g., neutrophils, macrophages, T cells) across a compromised blood-brain barrier (BBB) (Loane and Kumar, 2016). TBI-induced BBB disruption is a hallmark pathological event, allowing the uncontrolled entry of neurotoxic substances, inflammatory mediators, and cellular debris from the periphery into the delicate brain parenchyma, further contributing to widespread neuronal damage, dysfunction, and persistent inflammation. Activated microglia and astrocytes, initially beneficial for debris clearance, become dysregulated and release a plethora of pro-inflammatory cytokines (e.g., IL-1β, TNF-α), chemokines, and reactive oxygen species (Shen et al., 2022). This chronic inflammatory milieu creates a hostile microenvironment that significantly impairs synaptic plasticity, alters neurotransmission, and can induce widespread neuronal death. This sustained inflammatory state can profoundly alter brain network dynamics, contribute to the prolonged cognitive and emotional deficits seen in TBI survivors, and potentially predispose to or profoundly modify NDE experiences by altering neuronal excitability, neurotransmitter balance, and the brain’s overall homeostatic regulation. The long-term activation of glial cells also impacts myelination and white matter integrity, which can further disrupt neuronal communication across networks.

## Genetic and epigenetic modulators

Emerging evidence from neuropsychogenetics and epigenetics suggests that individual genetic predispositions and subsequent epigenetic modifications may significantly influence both susceptibility to altered states of consciousness and the propensity for post-traumatic creativity following TBI.

### COMT, BDNF, and DRD2 polymorphisms

#### COMT Val158Met polymorphism

This common single nucleotide polymorphism (SNP) in the catechol-*O*-methyltransferase (COMT) gene significantly affects dopamine metabolism, particularly in the prefrontal cortex. The Val allele leads to higher COMT activity and faster dopamine breakdown, while the Met allele results in lower activity and higher synaptic dopamine levels. This polymorphism is strongly linked to individual differences in cognitive flexibility, emotional regulation, and even spiritual perception (Klucken et al., 2015). Variations in dopamine signaling, modulated by COMT, could profoundly influence the brain’s rewards and salience processing pathways, potentially contributing to the profound positive emotional tone, sense of interconnectedness, and spiritual insights often reported in NDEs. The differential dopamine levels could also affect the intensity and valence of the experience.

#### BDNF Val66Met variation

This genetic variation in the brain-derived neurotrophic factor (BDNF) gene impacts the intracellular trafficking and secretion of BDNF, a crucial neurotrophin vital for neuronal survival, growth, differentiation, and especially synaptic plasticity and memory reconsolidation (Egan et al., 2003). Alterations in BDNF signaling, due to this polymorphism, could thus significantly influence the brain’s capacity to cope with traumatic injury, undergo adaptive rewiring (neuroplasticity), and integrate the profound and often life-changing insights gained from NDEs into long-term memory and personality. The Val66Met polymorphism has been linked to altered hippocampal function and memory (Egan et al., 2003; Hariri et al., 2003), which are critical for the narrative construction of NDEs.

#### Variants in DRD2

Polymorphisms in the dopamine receptor D2 (DRD2) gene have been specifically associated with individual variations in mystical experiences, spiritual beliefs, and altered rewards processing (Roy et al., 2021). Given the dopamine system’s central role in rewards, motivation, salience attribution, and its known involvement in various altered states of consciousness (e.g., psychosis), DRD2 variants make it a key candidate for mediating the powerful subjective experiences of euphoria, profound peace, and heightened perception often reported post-NDE. Differential sensitivity of D2 receptors could influence the subjective interpretation and emotional valence of the NDE.

We should also clarify that these polymorphisms (e.g., Val66Met) are pre-existing genetic predispositions that may moderate an individual’s response to TBI. We clearly distinguish these from post-injury epigenetic modifications (e.g., DNA methylation), which are induced by the trauma itself.

## Epigenetics of trauma and creativity

Beyond fixed genetic predispositions, severe trauma like TBI can trigger dynamic and enduring epigenetic modifications. This section now focuses solely on long-term changes. We propose a more specific mechanism: TBI-induced inflammation and chronic stress may trigger specific epigenetic modifications (e.g., in the promoters for BDNF or 5-HT receptor genes) that, in turn, “lock in” a state of heightened neuroplasticity or altered sensitivity, thereby contributing to the enduring creative and psychological shifts reported post-NDE. These reversible changes to gene expression, without altering the underlying DNA sequence, include alterations in DNA methylation (the addition of a methyl group to DNA) and histone acetylation (the modification of histone proteins around which DNA is wrapped) (Levenson and Sweatt, 2005; Woldemichael et al., 2014). These modifications can occur particularly in genes regulating stress response pathways (e.g., HPA axis genes), neuroinflammation, and critically, synaptic plasticity. Such TBI-induced epigenetic changes may facilitate long-term, fundamental shifts in emotional processing, cognitive function, and even personality, thereby creating a profound biological foundation for both spiritual transformation and the unexpected emergence of artistic or creative abilities. For instance, epigenetic regulation of genes involved in glutamatergic neurotransmission, neurotrophic factor expression, or specific neuronal receptor subtypes could lead to persistent alterations in neuronal excitability, circuit function, and overall network connectivity. This could influence both the acute NDE experience (e.g., by altering sensitivity to endogenous neurochemicals) and the remarkable long-term changes in personality, spiritual outlook, and creative expression observed post-trauma. The brain’s epigenetic landscape is highly plastic and can be profoundly shaped by extreme experiences, offering a molecular link between trauma and profound psychological and cognitive shifts.

## Evolutionary perspectives

From an evolutionary standpoint, NDEs and their intricate psychological aftermath may be framed as deep-seated, potentially adaptive cognitive responses to extreme, life-threatening stress. Theories propose that the dissociative phenomena often experienced during life-threatening events might function as an ancient evolutionary defense mechanism, allowing individuals to mentally detach from overwhelming physical pain, existential dread, or imminent fear (Xu et al., 2023). This “freeze,” “tonic immobility,” or “psychological escape” response, mediated by specific brain circuits involving the periaqueductal gray (PAG) and its connections to the limbic system, might be an evolutionarily conserved survival mechanism that, when triggered in modern contexts (like severe TBI), gives rise to the unique phenomenology of NDEs. We must clarify that the adaptiveness lies in the original “freeze” (thanatosis) response, which can cause a predator to lose interest, rather than in the NDE itself. We hypothesize that the NDE is the modern phenomenological experience of this ancient brainstem circuit (involving the PAG) being activated by the profound life-threat of a TBI. We propose that the intense physiological shock and profound fear from the TBI itself acts as the life-threatening trigger that activates this conserved PAG-mediated “freeze” circuit. This aligns with recent models of “thanatosis” as a key explanatory framework for core NDE features (Peinkhofer et al., 2021). This detachment could reduce the immediate physiological impact of trauma or allow for a more adaptive response in certain predator-prey scenarios. Furthermore, the intriguing “creative explosion” sometimes observed in TBI survivors, particularly those with NDEs, may reflect the unexpected unmasking of latent right-hemispheric capabilities. We elaborate on this “acquired savantism” hypothesis, positing that TBI-related damage, particularly to the left hemisphere, may “unmask” or disinhibit latent creative and cognitive capacities in the right hemisphere. This concept, explored in the work of McGilchrist (2019) and others, suggests a re-balancing of hemispheric contributions to cognition and perception. This phenomenon could echo evolutionarily conserved neural redundancies and alternative processing pathways, designed to enhance survival flexibility and problem-solving abilities under conditions of severe injury or threat to primary neural systems. These dormant or suppressed systems, particularly those associated with non-linear thinking, holistic perception, and artistic expression, may become remarkably accessible and dominant through injury-induced neuroplastic realignment. The injured brain, facing functional deficits in typical pathways, might re-route and activate alternative, previously underutilized neural circuits to compensate, inadvertently unlocking latent creative potential. This profound capacity for adaptive rewiring highlights the brain’s remarkable resilience and intrinsic drive toward functional recovery, even if it leads to unexpected forms of expression.

## Clinical cases and neuroimaging studies

Clinical research has provided compelling insights into the neurobiological underpinnings of NDEs. One pivotal clinical study by Parnia et al. (2014) meticulously examined patients who experienced cardiac arrest, another major cause of NDEs and a state of profound cerebral anoxia. This paradox, however, has been challenged and refined by recent findings. We must move past the “silent brain” model, as recent studies, such as the AWARE II trial and work by Borjigin et al. (2013), have detected surges of coherent EEG gamma activity in the dying brain. Our argument is now revised to hypothesize that the TBI-induced hypoxic brain, rather than being silent, may exhibit a similar surge of hyper-connected, organized activity, providing a more plausible neurobiological basis for complex, vivid NDEs. Despite neuroimaging data revealing significantly reduced or absent cerebral activity during the period of cardiac arrest, some patients reported detailed, “veridical” NDEs–i.e., structured perceptions of events that occurred outside their direct sensory field while they were clinically unconscious. These cases provide crucial evidence that complex, vivid experiences and aspects of consciousness may persist or emerge even in conditions of minimal or non-detectable brain activity, challenging conventional neuroscientific models that strictly equate consciousness with maximal cortical activity. The implications for TBI survivors are profound, suggesting that the brain, even when severely compromised by injury and facing profound energy deficits, may be capable of producing complex and highly structured subjective experiences. Another influential study by van Lommel et al. (2002) prospectively analyzed NDEs in a large cohort of cardiac arrest patients, finding that those who experienced NDEs reported significant and lasting increases in prosocial attitudes, spirituality, and appreciation for life compared to non-NDE survivors of cardiac arrest, underscoring the profound psychological changes. While conducted in cardiac arrest, these findings strongly suggest that similar profound alterations in regional cerebral blood flow and metabolic activity may occur in specific brain areas associated with self-perception and consciousness in TBI patients, potentially mediating the NDE. These studies underscore the necessity of moving beyond simple presence or absence of brain activity to understanding dynamic shifts in network states and the potential for residual or emergent consciousness under extreme physiological duress.

## The phenomenology of NDEs post-TBI

Traumatic brain injury survivors who experience NDEs frequently report transformative and deeply life-altering phenomena that can lead to profound, lasting changes in their worldview, core personality traits, and fundamental sense of self. These shifts are not merely superficial psychological adjustments but often manifest as a sustained increase in spirituality, a powerful sense of interconnectedness with others and the universe, and a significantly reduced or complete absence of the fear of death (Greyson and Stevenson, 1980; Greyson, 2000, 2006). Our understanding of TBI-specific NDEs is informed by the work of Hou et al. (2013), who found that NDEs in this cohort were infrequent but shared key features with NDEs from other etiologies, including many of the phenomena described below. Several interconnected psychological dimensions are critical to fully understanding these profound post-NDE transformations.

## Personality and spirituality changes

A highly recurrent and consistently reported theme in the phenomenology of NDEs following TBI is a marked and often permanent shift toward increased spirituality or a sense of existential meaning. For example, *Case 3*: John, a patient who sustained a severe TBI in a car accident, described a vivid NDE that involved encountering deceased loved ones and experiencing an overwhelming sense of peace and profound connection with the universe. Following his recovery, John reported a dramatic increase in his spiritual beliefs, a newfound sense of purpose in life, and a diminished focus on material possessions. However, he also articulated significant struggles with social reintegration and reconciling his altered perspectives with his pre-existing relationships and life goals (Greyson, 2022). This highlights the dual nature of NDEs: while they can foster deeply positive psychological growth, they also present significant challenges in terms of psychological adjustment and the difficult process of integrating radically altered beliefs and values with a pre-existing social and personal framework. The profound shift in values can sometimes lead to feelings of alienation or difficulty relating to others who have not shared similar experiences.

## Cognitive and emotional shifts

Beyond spiritual transformations, TBI survivors who experience NDEs may also undergo significant and often complex changes in their cognitive and emotional processing. These changes can include subtle to moderate impairments in memory encoding and retrieval, reduced attentional capacity, and fundamentally altered emotional responses (Weiler et al., 2024). While some survivors report heightened emotional sensitivity, increased empathy, or a greater capacity for compassion, others struggle with debilitating post-traumatic stress disorder (PTSD) symptoms, including intrusive flashbacks or persistent, distressing intrusive memories directly related to their injury and the NDE. (This case is an illustrative composite). *Case 4*: Laura, another TBI survivor, reported vivid, recurrent flashbacks to her NDE experience, in which she vividly encountered deceased family members. These flashbacks were frequently accompanied by intense emotional reactions, including anxiety and dysphoria, and periods of disorientation, particularly in situations that reminded her of the accident or the NDE itself. The significant psychological burden imposed by these intrusive experiences underscores the critical need for comprehensive and tailored therapeutic interventions, such as trauma-focused cognitive behavioral therapy (CBT) or eye movement desensitization and reprocessing (EMDR), following NDEs to facilitate healthy integration and prevent chronic psychological distress.

## The life review

We include this core phenomenon here to provide clear signposting: understanding the “Life Review” is essential for linking it to a TBI-specific mechanism (e.g., trauma-induced temporal lobe activation) and for understanding how it leads to long-term change (e.g., the reframing of one’s personal narrative, which can catalyze new creative expression).

## The emergence of artistic abilities post-TBI

One of the most striking and neurologically intriguing phenomena reported by a subset of NDE survivors is the unexpected and often dramatic emergence of artistic abilities, particularly in the realms of music, visual arts (e.g., painting, sculpture), and creative writing. These abilities often appear abruptly, with little to no prior training or interest, and may be described as savant-like - a term used to describe extraordinary talents that emerge following brain injury, even in individuals who had no prior interest or discernible skill in the domain (Miller et al., 1998). *Case 5*: Tony Cicoria, whose NDE after a lightning strike was mentioned earlier, is a prime and extensively documented example of a TBI survivor who experienced an explosion of musical creativity. Cicoria’s newfound and profound ability to compose and perform complex classical music, despite having no prior musical background or formal training, suggests a profound and fundamental shift in his neural architecture and functional connectivity following the trauma. This phenomenon can be eloquently explained by the concept of neuroplasticity, the brain’s extraordinary intrinsic capacity to reorganize its neural circuits and form new synaptic connections in response to injury, damage, or novel experiences (Miller et al., 1998). Another equally striking example is *Case 6*: Vladimir, a TBI survivor who experienced a significant and unexpected transformation in his artistic abilities after surviving a severe head injury sustained in a fall. Prior to his accident, Vladimir had no background or interest in art, but following his remarkable recovery, he began spontaneously producing highly detailed, technically precise, and emotionally evocative portraits that garnered considerable attention for their profound emotional depth and striking technical precision. His case robustly exemplifies how brain injury, in rare and fascinating instances, can paradoxically unlock latent creative potential, reshaping cognitive landscapes in unexpected ways.

## Neurobiological mechanisms of artistic emergence

The precise neurological mechanisms underlying the emergence of artistic abilities in TBI survivors remain an active and complex area of scientific inquiry. However, several compelling theories point to the converging roles of extensive neuroplasticity, functional disinhibition of specific brain regions, and the critical involvement of the right cerebral hemisphere in this transformative process. Following TBI, especially if the primary injury involves the left cerebral hemisphere (which typically dominates verbal and logical processing), compensatory activation and enhanced processing in the right hemisphere - traditionally associated with artistic, creative, holistic, and non-linear abilities - may occur. This increased reliance on the right hemisphere can lead to the “unmasking” of latent creative talents that were previously suppressed or underexpressed (Treffert, 2006). This phenomenon is often categorized as acquired savant syndrome, a rare but profound condition in which previously dormant or nascent abilities are dramatically activated after brain injury (Miller et al., 1998).

The right hemisphere, particularly cortical areas like the right anterior temporal lobe (rATL), the right inferior frontal gyrus (rIFG), and specific parietal lobe regions, plays a central and well-established role in creative ideation, visual-spatial processing, musicality, and metaphoric language (Flaherty, 2005). Damage to the left hemisphere or its typical inhibitory pathways to the right hemisphere may result in the profound disinhibition of these right-hemispheric functions, leading to the spontaneous and often prolific expression of artistic abilities that were previously suppressed, unexplored, or simply lacked the necessary neural “release” (Miller et al., 1998). We elaborate on this “acquired savantism” hypothesis, positing that TBI-related damage, particularly to the left hemisphere, may “unmask” or disinhibit latent creative and cognitive capacities in the right hemisphere. This concept, explored in the work of McGilchrist (2019) and others, suggests a re-balancing of hemispheric contributions to cognition and perception. This disinhibition is often facilitated by altered resting-state network connectivity, where previously suppressed or less active networks become significantly more active and influential following injury. At a more granular cellular and molecular level, the profound neuroplasticity observed post-TBI, which underpins both recovery and unexpected skill emergence, involves a cascade of intricate mechanisms:

### Axonal sprouting and synaptogenesis

In response to neuronal loss or damage, surviving neurons can form new axonal branches (sprouting) and create new synaptic connections (synaptogenesis) to compensate for lost inputs or re-establish disrupted circuits (Carmichael, 2006). This reorganization can lead to novel functional pathways.

### Changes in dendritic spine morphology

The structure and density of dendritic spines, the tiny protrusions on dendrites where most excitatory synapses occur, are highly dynamic. TBI can induce changes in spine morphology, leading to altered synaptic strength, efficiency, and overall network excitability, which can be critical for learning and new skill acquisition (Ehrlich and Malinow, 2004).

### Adult neurogenesis

While limited in the adult mammalian brain, the generation of new neurons (neurogenesis) primarily occurs in the subgranular zone of the hippocampus and the subventricular zone (Gage, 2000). While its direct contribution to functional recovery and savant syndrome is still under intense investigation, enhanced neurogenesis in response to injury could theoretically contribute to neural reorganization.

### Modulation of neurotransmitter receptor expression and sensitivity

Traumatic brain injury can alter the number, localization, and sensitivity of various neurotransmitter receptors (e.g., glutamate, GABA, dopamine receptors) on neuronal membranes. These changes can significantly alter neuronal excitability, responsiveness to incoming signals, and the overall balance of excitation and inhibition within neural circuits, potentially unlocking latent creative potential (Mattson, 2008).

### Glial cell remodeling and interaction with neurons

Astrocytes and microglia, initially crucial for the acute inflammatory response and debris clearance, undergo long-term remodeling post-TBI. Beyond their immune roles, these glial cells actively participate in synaptic pruning, promoting or inhibiting new synapse formation, modulating neurotransmitter levels, and influencing the overall function and plasticity of neuronal circuits (Fields and Richardson, 2019; Colonna and Butovsky, 2017). Their long-term dysregulation or adaptive remodeling post-TBI can profoundly contribute to altered network function and the emergence of new cognitive abilities. For instance, some glia release specific growth factors that promote neuronal survival or synaptic remodeling.

These underlying molecular and cellular changes contribute to a fundamental neuroplastic realignment where the brain, in its extraordinary attempt to recover from injury and adapt to new challenges, may reallocate computational resources, reconfigure existing circuits, and paradoxically “unmask” or facilitate the expression of previously dormant capabilities. This dynamic process highlights the brain’s inherent resilience and adaptability.

## Psychological and existential shifts

Survivors of NDEs frequently report profound, long-lasting transformations. For example, the prospective study by van Lommel et al. (2002) found that NDE experiencers reported significant and lasting increases in prosocial attitudes, spirituality, and appreciation for life compared to non-NDE survivors of cardiac arrest, underscoring the profound psychological changes. In addition to the demonstrable neurological mechanisms, the profound psychological and existential transformation that many TBI survivors undergo following NDEs is also critically important in comprehensively understanding the emergence of artistic abilities. Many individuals who experience NDEs report a deep, ineffable sense of connection to the universe, a heightened appreciation for life, a profound shift in values, and a newfound, often overwhelming desire to express these intense feelings and insights through artistic creation (Bicego et al., 2023). For example, *Case 7*: Lara, a survivor of a traumatic brain injury and subsequent NDE, spontaneously began painting after her experience, creating deeply evocative and symbolic works that explored themes of universal love, spiritual connection, the interconnectedness of all life, and profound spirituality. Her art became not just an aesthetic pursuit but a vital means of processing, integrating, and communicating the profound experiences and revelatory insights gained during her NDE. This compelling phenomenon suggests that artistic expression post-NDE may not solely be a direct result of underlying neurobiological changes (e.g., disinhibition), but also a potent and essential form of psychological healing, self-expression, and a means to assimilate a transformative experience into one’s evolving identity and worldview. It is a way to make sense of the incomprehensible and share newfound meaning.

## Additional case reflections and artistic neurocognition

The following cases are illustrative composites based on common themes in the literature, as many single published reports are anonymized. Where possible, we have replaced these with citations to actual documented case reports (e.g., Miller et al., 1998; Sacks, 2007). To better illustrate the full trajectory from injury to creative emergence, we can also draw from the “acquired savantism” literature (e.g., Treffert, 2010), which often has a TBI etiology.

*Case 8*: Alan, a previously academically focused 19-years-old TBI patient, unexpectedly began composing highly intricate and emotionally resonant poetry, richly imbued with metaphysical imagery and existential themes, following a near-fatal motor vehicle accident and associated NDE. Functional magnetic resonance imaging (fMRI) performed post-recovery revealed hyperactivation in his right anterior temporal and right parietal cortices during poetic composition, areas typically associated with semantic processing, metaphor generation, and creative insight (Liu et al., 2024); *Case 9*: Sofia, a middle-aged woman with a history of diffuse axonal injury (a common TBI pathology affecting white matter tracts), developed a sudden and remarkable aptitude for oil painting, despite no prior artistic inclination or training. Her works often depicted recurring motifs of luminous tunnels, brilliant light sources, and ethereal figures, strikingly suggestive of her profound NDE vision. These visual themes serve as direct translations of her subjective experience into artistic form. These cases collectively highlight the probable involvement of the right inferior frontal gyrus, right anterior temporal lobe, and various parietal lobe regions, areas consistently linked to various aspects of creative ideation, visual-spatial processing, and the generation of metaphoric language (Flaherty, 2005; Fink et al., 2009). The specific types of creativity that emerge may depend on which of these regions are preferentially disinhibited or reorganized.

## Neuromodulatory and pharmacological interventions: unpacking consciousness and creativity

While direct interventions to induce NDEs or specific forms of post-TBI creativity are not ethically or scientifically pursued, studying the effects of neuromodulatory and pharmacological agents offers invaluable theoretical insights into the neural circuits and neurochemical pathways potentially involved in these phenomena. Investigating how these interventions alter consciousness, perception, and cognitive function can provide indirect clues about the neurobiology of NDEs and acquired savant syndromes.

## Deep brain stimulation (DBS)

Deep Brain Stimulation (DBS) involves surgically implanting electrodes into specific brain targets, which then deliver continuous, high-frequency electrical impulses. Primarily used for movement disorders like Parkinson’s disease and essential tremor, and increasingly for psychiatric conditions such as severe obsessive-compulsive disorder (OCD) and depression, DBS works by modulating abnormal neuronal activity within dysfunctional brain circuits (Perlmutter and Mink, 2006).

## Theoretical relevance to NDEs and creativity

### Consciousness alterations

While rare, some patients undergoing DBS for neurological or psychiatric conditions have reported transient altered states of consciousness, including feelings of detachment, vivid memories, or even brief dissociative episodes, depending on the stimulation target (Schlaepfer et al., 2014). For instance, stimulation of limbic areas (e.g., subthalamic nucleus, ventral striatum) or specific cortical targets could potentially influence neural circuits implicated in self-awareness, emotional processing, and memory retrieval, which are central to NDE phenomenology. If NDEs involve a “reset” or disinhibition of specific brain networks, then DBS, by precisely modulating network activity, could theoretically offer a controlled way to explore how these networks contribute to consciousness.

### Memory and experience re-activation

Stimulation of certain temporal lobe structures or deep brain nuclei has been shown to induce vivid autobiographical memories or dream-like states (Mankin and Fried, 2020). This could provide a model for understanding the “life review” aspect of NDEs, where memories are rapidly accessed and processed.

### Creativity and affect

Some anecdotal reports suggest changes in mood, personality, or even creative output in DBS patients, though this is often a complex interplay of disease effects, medication, and stimulation (Drago et al., 2009). For example, DBS in the subthalamic nucleus for Parkinson’s disease, by modulating dopamine pathways, has been reported to affect rewards sensitivity, motivation, and sometimes lead to the emergence of artistic hobbies or hypergraphia in rare cases, albeit not in the “savant” sense. If creativity involves the disinhibition of certain brain circuits or altered dopamine function, DBS could offer a tool to investigate these pathways. However, DBS is a highly invasive procedure, and its application to “induce” or “enhance” NDEs or creativity is currently beyond ethical and practical considerations. Its primary relevance lies in providing insights into circuit function.

## Transcranial magnetic stimulation (TMS)

Transcranial Magnetic Stimulation (TMS) is a non-invasive neuromodulation technique that uses rapidly changing magnetic fields to induce electrical currents in targeted areas of the brain, thereby modulating neuronal activity (Rossi et al., 2009). Depending on the frequency and intensity, TMS can either increase or decrease cortical excitability. It is clinically approved for depression, OCD, and migraine, among others.

### Theoretical relevance to NDEs and creativity

#### Out-of-body experiences (OBEs)

As mentioned earlier, early research by Blanke et al. (2002) demonstrated that TMS applied to the temporo-parietal junction (TPJ) could reliably induce sensations of an out-of-body experience in healthy individuals. This direct experimental evidence strongly suggests that the TPJ is a critical neural correlate for self-location and body perception. Further TMS studies targeting the TPJ or related areas could potentially refine our understanding of the specific neural processes that become perturbed during NDEs, leading to dissociative phenomena.

#### Disinhibition and creativity

Repetitive TMS (rTMS) can induce temporary “virtual lesions” or enhance activity in specific cortical regions. Low-frequency rTMS applied to the left anterior temporal lobe (a region implicated in semantic memory and rule-based processing) has been shown to temporarily enhance certain aspects of creativity, such as insight problem-solving or artistic abilities, in healthy individuals (Salvi et al., 2020; Daskalakis et al., 2006). The hypothesis is that transiently inhibiting a dominant, left-hemisphere-mediated cognitive style might disinhibit the right hemisphere’s more holistic or creative processing. This mirrors the “disinhibition hypothesis” proposed for acquired savant syndrome post-TBI. TMS could therefore be a valuable research tool to mimic aspects of TBI-induced disinhibition in a controlled, reversible manner, helping to elucidate the neural underpinnings of artistic emergence.

#### Altered perception and affect

Transcranial magnetic stimulation applied to prefrontal or parietal regions can alter mood, attention, and perception, providing a window into how altered neural activity in specific circuits can influence subjective experience (Rossi et al., 2009). This could contribute to understanding the emotional and perceptual shifts seen in NDEs.

#### Ketamine effects

Ketamine is a dissociative anesthetic and increasingly recognized as a rapid-acting antidepressant. Its primary mechanism of action involves non-competitive antagonism of the NMDA receptor, a type of glutamate receptor (Zarate et al., 2006). At sub-anesthetic doses, ketamine produces profound dissociative states, altered perceptions, vivid hallucinations, and a unique sense of ego dissolution or “mystical experience.”

### Theoretical relevance to NDEs and creativity

Ketamine’s ability to induce dissociative states strongly aligns with the hypothesis that alterations in glutamatergic signaling, particularly involving NMDA receptors, are central to the dissociative aspects of NDEs (Jansen, 1997; Krystal et al., 1994). TBI often leads to excitotoxicity mediated by excessive glutamate and NMDA receptor activation, but the complex interplay of overactivation, subsequent downregulation, or receptor damage could produce various states, including dissociation. Ketamine provides a pharmacological model to study these effects in a controlled setting. The out-of-body sensations, feelings of unreality, and altered body schema reported with ketamine bear striking resemblances to NDE reports. Higher doses of ketamine can induce a profound sense of ego dissolution, feelings of unity, and a sense of transcending physical boundaries, which are core features of many NDEs and mystical experiences (Fritz et al., 2025). This suggests that pharmacological modulation of NMDA receptors can directly access neural pathways that mediate these transcendental states, offering a unique opportunity to study their neurochemical basis.

#### Neuroplasticity and synaptogenesis

Emerging research indicates that ketamine, particularly in its antidepressant effects, can rapidly induce synaptogenesis and enhance neuroplasticity, especially in the prefrontal cortex (Duman et al., 2016). This pro-plasticity effect, mediated partly by mTOR signaling pathways, could be conceptually linked to the brain’s capacity for reorganization post-TBI and the emergence of new abilities. While speculative, if ketamine promotes adaptive neuroplasticity, it could theoretically, in certain contexts, modulate the brain’s capacity for novel functional organization, although its direct role in specific artistic emergence is not established.

#### Altered perceptual processing

Ketamine affects sensory gating and integration, leading to distorted perceptions of time, space, and self (Anticevic et al., 2016). These effects mirror the time distortion and altered reality perception frequently reported in NDEs.

It is critical to emphasize that while DBS, TMS, and ketamine offer valuable tools for probing neural circuits involved in consciousness and perception, their direct role in either causing NDEs or reliably enhancing creativity in TBI survivors remains theoretical and subject to extensive future research. These interventions are primarily used for therapeutic purposes in specific patient populations, and their application to investigate these complex phenomena would require rigorous ethical oversight and scientific justification. Nevertheless, they provide unique lenses through which to understand the neurobiological underpinnings of extraordinary brain states.

## Bridging neuroscience, consciousness, and creativity

The intricate intersection of NDEs, traumatic brain injury, and the unexpected emergence of artistic abilities offers a profoundly fascinating and fertile ground for scientific inquiry, providing unparalleled insights into the brain’s remarkable capacity for both fundamental transformation and astonishing recovery following severe injury. By rigorously exploring the neurobiological, phenomenological, and psychological aspects of these deeply complex phenomena, we gain invaluable insights into the very nature of human consciousness, the mechanisms of creativity, and the extraordinary resilience and adaptability of the human mind. Clinical case studies, meticulously detailed patient reports, and advanced neuroimaging findings collectively suggest that the brain can produce exceptionally profound and structured subjective experiences, including comprehensive NDEs, even under conditions of severe physiological duress and significant structural or functional compromise. Moreover, the recurrent emergence of artistic abilities following TBI and NDEs powerfully underscores the remarkable and untapped potential of the human brain for rapid neuroplastic adaptation and the astonishing unmasking of latent talents. As cutting-edge research into this enigmatic area continues to progress, we can anticipate further groundbreaking advancements in understanding the complex, reciprocal relationship between brain injury, altered states of consciousness, and the genesis of creativity. These advancements will have significant and far-reaching implications for the development of innovative neuropsychological rehabilitation strategies, the application of art therapy in neurorecovery, and fundamentally, our broader philosophical and scientific understanding of the brain’s intrinsic adaptive capacities. Future research integrating multi-omics approaches (including genomics, transcriptomics, proteomics, and metabolomics) will be essential to unraveling the molecular signatures associated with NDEs and post-TBI creativity. Such approaches could identify specific genetic predispositions, gene expression changes, protein modifications, and metabolic shifts that underpin these phenomena. Furthermore, detailed connectomics - the comprehensive mapping of brain connectivity at various scales, from microcircuits to large-scale networks - will be critical to precisely characterize the dynamic alterations in neural network architecture that give rise to NDEs and facilitate artistic emergence. Finally, longitudinal psychological profiling combined with neuroimaging and omics data will be crucial for understanding the long-term trajectories of these experiences and their impact on individuals’ lives. These integrated insights promise not only to redefine the neurobiological narrative of recovery from TBI but also to profoundly challenge our fundamental understanding of identity, the very essence of consciousness, and the brain’s infinite capacity for renewal and self-transcendence. Specifically, we recommend future systematic reviews and meta-analyses to quantify the prevalence of NDEs in TBI cohorts and to statistically assess the strength of the association between NDEs and subsequent creative enhancements. Furthermore, comparative studies of NDE-like experiences in TBI versus neurodegenerative diseases, such as Alzheimer’s, could provide crucial insights into the underlying mechanisms and phenomenological differences.

## Data Availability

The original contributions presented in this study are included in this article/supplementary material, further inquiries can be directed to the corresponding author.
